# Effect of Ruthenium and Cerium Oxide (IV) Promotors on the Removal of Carbon Deposit Formed during the Mixed Methane Reforming Process

**DOI:** 10.3390/ma14247581

**Published:** 2021-12-09

**Authors:** Mateusz Zakrzewski, Oleksandr Shtyka, Radoslaw Ciesielski, Adam Kedziora, Waldemar Maniukiewicz, Natalia Arcab, Tomasz Maniecki

**Affiliations:** Department of Chemistry, Institute of General and Ecological Chemistry, Lodz University of Technology, Zeromskiego 116, 90-924 Lodz, Poland; oleksandr.shtyka@p.lodz.pl (O.S.); radoslaw.ciesielski@p.lodz.pl (R.C.); adam.kedziora@p.lodz.pl (A.K.); waldemar.maniukiewicz@p.lodz.pl (W.M.); 231410@edu.p.lodz.pl (N.A.); tomasz.maniecki@p.lodz.pl (T.M.)

**Keywords:** methane reforming, ruthenium, cerium (IV) oxide, gasification, carbon deposit

## Abstract

This work investigates the effect of the addition of Ru and CeO_2_ on the process of gasification of carbon deposits formed on the surface of a nickel catalyst during the mixed methane reforming process. Activity studies of the mixed methane reforming process were carried out on (Ru)-Ni/CeO_2_-Al_2_O_3_ catalysts at the temperature of 650–750 °C. The ruthenium-promoted catalyst exhibited the highest activity. Carbonized post-reaction catalyst samples were tested with the TOC technique to investigate the carbonization state of the samples. The bimetallic catalyst had the lowest amount of carbon deposit (1.5%) after reaction at 750 °C. The reactivity of the carbon species was assessed in mixtures of oxygen, hydrogen, carbon dioxide, and water. Regardless of the gasifying agent used, the carbon deposit was removed from the surface of the catalytic system. The overall mechanism of mixed methane reforming over Ru and CeO_2_ was shown.

## 1. Introduction

The mixed methane reforming process discussed in this manuscript is a combination of two processes: (1) dry (CH_4_ + CO_2_ ⇌ 2CO + 2H_2_) and (II) steam methane reforming (CH_4_ + H_2_O ⇌CO + 3H_2_). In the research, biogas was used as the feed gas. It is produced by the fermentation of molasses in sugar factories. The resulting mixture consists of CH_4_:CO_2_:H_2_ in a 2:2:1 volumetric ratio [1,2].

It is well known that the catalyst used in the processes of methane reforming can undergo deactivation due to various effects, including (1) plugging micro- and mesopores, (2) blocking active sites by adsorbed forms, (3) encapsulating metal crystals by polymeric carbon (4), and destroying the catalyst structure by the formation of whiskers. Among them, the deactivation of catalysts due to the formation of carbon species on their surface is the most common.

Carbonized species can be formed as a result of the Boudouard reaction [3] and the decomposition of methane [4]. It is known that carbon monoxide can decompose into carbon and carbon dioxide. This reaction is exothermic and is favored by a lack of hydrogen.

2CO ⇌ CO_2_ + C→ H_R°298K_ = −172.5 kJ/mol (1)

Any carbon-containing molecule can become a carbon source, such as the methane decomposition shown in Equation (2).

CH_4_→ C + 2H_2_→ ΔH_R°298K_ = 74.9 kJ/mol (2)

Removal of the formed carbon deposit is associated with Reactions (3)–(8), i.e., gasification with gases such as H_2_, H_2_O, CO_2_, or O_2_.

C + 2H_2_ → CH_4_ (3)

C + H_2_O→ CO + H_2_ (4)

C + 2H_2_O→ CO_2_ + 2H_2_ (5)

C + CO_2_ ⇌ 2CO (6)

C + O_2_ → CO_2_ (7)

C + 1/2O_2_ → CO (8)

Depending on the structure of the carbon, the rate of catalyst regeneration is slow when there is no access to the nickel surface, or it may be faster as soon as the nickel surface is available to gasification agents. Regeneration of partially blocked nickel particles is easier than removing encapsulated carbon deposits or plugged pores. The latter forms of carbon deposit require more harsh conditions for their regeneration due to the lack of access to the active sites [5,6,7].

Regeneration of catalytic systems containing a carbon deposit leads to production losses due to interruptions caused by the regeneration process and, consequently, increased operating costs [8]. Another problem during the regeneration process at elevated temperatures is the sintering of the catalysts. Therefore, rather than removing the effects of deactivation, it is important to prevent carbon deposit formation.

The problem of catalyst deactivation as a result of carbon deposition is the competition between carbon formation and its gasification in situ. When the rate of gasification of the formed carbon deposit exceeds the rate of carbon formation, then the deactivation of the catalyst due to its carbonization can be prevented.

The probability of the formation of a carbon deposit during the methane reforming process depends not only on the partial pressures of individual reactants but also on the composition of the catalyst and the process conditions. For this purpose, various catalyst additives are used, e.g., magnesium, calcium, or potassium oxides, which means that the carbon deposit can be gasified faster than it is formed. Recently, cerium (IV) oxide has been used as an additive to the catalytic support (aluminum oxide) [9,10,11,12]. The Ce-based materials owe their advantages to rapid change in the oxidation state of Ce^4+^ ⇌ Ce^3+^, which leads to the release of oxygen and conversely to store oxygen in the ceria-based structure [9,10,11,12].

Nickel catalysts in methane reforming show high activities, but their problem is inactivation, especially due to the formation of a carbon deposit. To avoid this problem, the active phase is doped with noble metals such as Pt, Pd, Rh, or Ru. In particular, ruthenium has recently been widely used as an additive to nickel, which means that bimetallic (Ni-Ru) catalysts can be characterized by higher conversion and resistance to deactivation, especially to the formation of a carbon deposit, than monometallic catalysts [13,14,15,16,17,18,19].

This work aimed at investigating the effect of CeO_2_ and Ru addition on the formation of a carbon deposit in the mixed methane reforming process. It was especially interesting to study the concentration of inactive carbon and its reactivity to oxygen, hydrogen, carbon dioxide, and water vapor, participation of CH_4_ and CO_2_ activation pathways in the total carbon formation on the catalytic surface through methane decomposition and Boudouard reactions, and the OSC properties of CeO_2_ towards carbon deposit removal.

## 2. Materials and Methods

### 2.1. Catalyst Preparation 

Alumina was obtained by precipitation of aluminum hydroxide from an aqueous Al(NO_3_)_3_ solution with the use of ammonia as a precipitating agent. The precipitation process was carried out at the temperature of 80 °C by adding ammonia to the nitrate solution until the pH of the solution changed from acidic to basic (pH = 9–10). The precipitated Al(OH)_3_ was aged for 24 h, then filtered and washed with deionized water until the solution reached pH = 7. The obtained precipitate was dried at 100 °C for 1 h and calcined in a stream of oxygen at 500 °C for 4 h.

CeO_2_-Al_2_O_3_ support was obtained using cerium (V) nitrate. The aqueous nitrate solution was added dropwise to the previously obtained alumina support. Then, the resulting solution was left for 24 h. After evaporation of the solvent, the solid residue was dried at 100 °C for 1 h and then calcined in air at 500 °C for 4 h. The nominal loading of CeO_2_ was 5%. 

The monometallic Ni catalyst was prepared using the incipient wetness impregnation method. A nickel phase was deposited on CeO_2_-Al_2_O_3_ from an aqueous solution of nickel (II) nitrate and left for 24 h for impregnation. After the solvent was evaporated, the obtained catalyst was dried at 100 °C and then calcined at 500 °C. The nominal metal content in the obtained catalyst was 20%.

The bimetallic catalyst Ru-Ni was obtained by subsequent impregnation using an aqueous solution of ruthenium (III) chloride. The impregnation procedure was similar as reported before. The nominal Ru content in the Ru-Ni catalyst was 1%.

### 2.2. Methods and Instruments

The specific surface area of catalyst and support was measured on a Sorptomatic 1900 automatic instrument (Carlo Erba Instruments, Cornaredo, Italy) using the low-temperature adsorption of nitrogen. The specific surface areas of the catalyst samples were estimated from the measured monolayer capacity by the Brunauer–Emmett–Teller method. The Dollimore and Heal method was used to calculate the pore size distribution.

SEM–EDS analysis of catalyst after reduction was conducted in an S-4700 electron microscope (Hitachi, Tokyo, Japan), equipped with an energy dispersive spectrometer EDS (ThermoNoran, Madison, WI, USA).

TPR-H_2_ and TPR-CH_4_ tests were carried out in the AMI-1 apparatus manufactured by Altamira Instruments (Pittsburgh, PA, USA) using a reducing mixture of 5%H_2_-95%Ar or 5%CH_4_-95%He with a gas flow rate of 40 cm^3^/min. The mass of the samples of the tested catalytic systems was about 0.1 g, and the measurements were carried in the temperature range from room to 900 °C with a linear increase of 10 °C/min. Hydrogen consumption was measured using a thermal conductivity detector, and the TPR-CH_4_ measurements were monitored with a mass spectrometer detector. Prior reducibility measurements all investigated catalysts were oxidized in situ to remove any impurities on their surface. 

The content of total organic carbon in the catalytic samples after the mixed methane reforming process was determined using a TOC 5000A analyzer (Shimadzu, Kyoto, Japan). A nondispersive infrared gas analyzer was used as a detector for the detection of CO_2_ generated during carbon burning. The apparatus was calibrated with pure glucose.

The temperature-programmed surface reactions (TPSRs) were performed to investigate the susceptibility of carbon deposits formed on the surface of the catalyst during mixed methane reforming to gasification in various atmospheres. The TPSR measurements were carried out in a quartz reactor in the temperature range of 25–900 °C with a heating rate of 10 °C/min. In each test, about 0.1 g of catalyst was placed in a microreactor and was heated in an Ar atmosphere at 100 °C for 1 h. Next, the inert gas was switched either to reducing (5%H_2_-95%Ar, 100%H_2_, 5%CO_2_-95%Ar) or oxidizing (5%H_2_O-95%Ar and 5%O_2_-95%Ar) gas mixtures. The volumetric flow of the reaction gases was 30 cm^3^/min. The evolution of the gaseous products was analyzed as a function of temperature using a mass spectrometer.

The reactions of methane decomposition and carbon monoxide disproportionation were carried out on 20%Ni/5%CeO_2_-95%Al_2_O_3_ and 1%Ru-20%Ni/5%CeO_2_-95%Al_2_O_3_ catalysts in the temperature range from 25 to 900 °C with a linear heating rate of 10 °C/min. A sample of the catalyst (about 0.1 g) was placed in a quartz reactor and reduced in situ with a stream of hydrogen (5%H_2_-95%Ar) at 900 °C for 1 h. Then, the sample was exposed to methane (5%CH_4_-95%He) or carbon monoxide (5%CO-95%Ar) streams with a volumetric flow of 30 cm^3^/min. The reaction products were analyzed with a mass spectrometer detector (Hidden Analytical, Livonia, MI, USA).

Tests of catalytic activity in the mixed methane reforming reaction were carried out in a flow-type quartz microreactor under atmospheric pressure in the temperature range of 650–750 °C. The composition of the reaction mixture in each test was as follows: CH_4_:CO_2_:H_2_:H_2_O:Ar = 2:2:1:0.9:1.25 (volumetric ratio). The total flow of the reaction mixture was 100 cm^3^/min. In each test, about 0.4 g of catalyst was placed in the reactor. The catalytic activity was measured after the system was stabilized for 3 h. The gas composition before and after the reaction was measured using gas chromatography with thermal conductivity detectors and two chromatographic columns: (I) Molecular Sieve 5A column (Restek, Bellefonte, PA, USA) and (II) Shin Carbon ST packed column (Restek, Bellefonte, PA, USA). 

Methane and carbon dioxide conversions were calculated using the following formulas:

(9) $$K_{{\mathrm{CH}}_{4}}=\left(\frac{W_{0{\mathrm{CH}}_{4}}-\left(W_{{\mathrm{iCH}}_{4}}\times \frac{W_{0\mathrm{Ar}}}{W_{\mathrm{iAr}}}\right)}{W_{0{\mathrm{CH}}_{4}}}\right)\times 100\%$$

(10) $$K_{{\mathrm{CO}}_{2}}=\left(\frac{W_{0{\mathrm{CO}}_{2}}-\left(W_{{\mathrm{iCO}}_{2}}\times \frac{W_{0\mathrm{Ar}}}{W_{\mathrm{iAr}}}\right)}{W_{0{\mathrm{CO}}_{2}}}\right)\times 100\%$$

where W_i_CH_4_, W_i_CO_2_, and W_i_Ar correspond to the average content of CH_4_, CO_2_, or Ar from three surface measurements originating from the injection of the reaction mixture at a given temperature, and W_0_CH_4_, W_0_CO_2_, and W_0_Ar correspond to the average of the CH_4_, CO_2_, or Ar standard from three surface measurements originating from the injection of the standard mixture.

## 3. Results

### 3.1. The Specific Surface Area of the Catalytic Materials

The specific surface area (SSA) and pore radius for nickel are given in Table 1. The specific surface area of the support was found to decrease from 170 to 147 m^2^/g after deposition of nickel or nickel and ruthenium particles. This is related to a high concentration of low-surface-area metal phases. According to the X-ray diffraction (XRD) measurements, the average size of Ni crystallites was about 45 and 16 nm for mono- and bimetallic catalyst systems, respectively. The average size of metal particles was calculated using the Scherrer equation. The analysis of the metal content of both catalysts is in good agreement with the calculations (Table 1).

### 3.2. Morphology Studies

**Figure 1:**
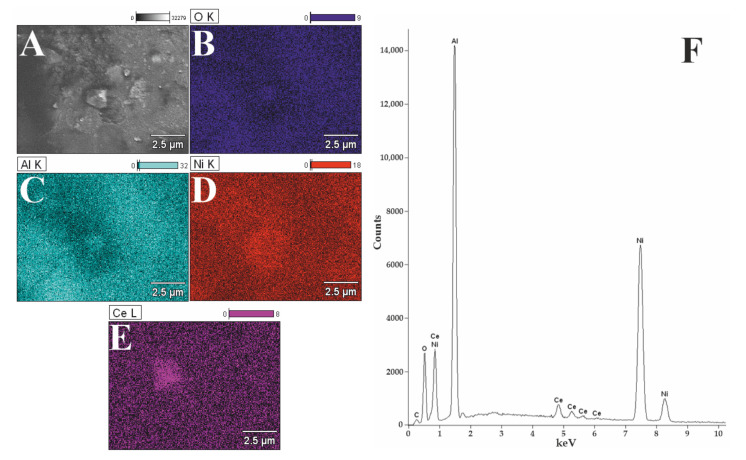
SEM–EDS image (**A**), EDS elemental mapping (**B**–**E**), and EDS spectrum (**F**) recorded for reduced bimetallic 1%Ru-20%Ni/5%CeO_2_-95%Al_2_O_3_ catalyst.

The 1%Ru-20%Ni/5%CeO_2_-95%Al_2_O_3_ was analyzed by scanning electron microscopy with an energy dispersive spectrometer. SEM–EDS was used to characterize the morphology and determine the elemental composition of the surface of the bimetallic system after reduction at 750 °C (Figure 1). The analysis of the surface composition of the bimetallic catalyst showed the presence of nickel, aluminum, cerium, and oxygen in the investigated system. We can observe that the location of the Al and O element can overlap each other (Figure 1B,C). Nickel (Figure 1D) and cerium (Figure 1E) are evenly distributed despite the presence of various structures on the surface. Even though the catalyst contains ruthenium, this element is invisible due to its low content and low detection threshold of the apparatus or because it can form an alloy with nickel, and such a low ruthenium content is obscured by a high nickel content [20], which is also confirmed by the EDS spectrum (Figure 1F).

In order to check the presence of the Ru-Ni alloy, Mierczynski et al. performed an XRD test for a catalyst with 5% Ru content. The conducted research shows that the Ru-Ni alloy is formed, which increases the catalytic activity of the tested system. Additionally, Mierczynski found that if the Ru-Ni alloy is formed with 5% ruthenium content in the catalyst, it will also form in the catalytic system containing 1% Ru [21].

### 3.3. Reduction Studies (TPR–H_2_)

**Figure 2:**
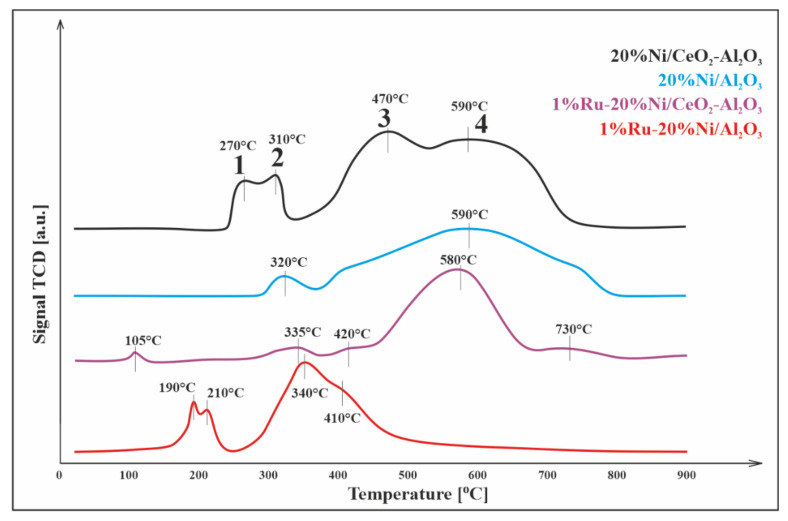
TPR-H_2_ profiles of 20%Ni/5%CeO_2_-95%Al_2_O_3_, 20%Ni/Al_2_O_3_, 1%Ru-20%Ni/5%CeO_2_-95%Al_2_O_3_, and 1%Ru-20%Ni/Al_2_O_3_ catalysts.

The reducibility of catalysts was examined using temperature-programmed reduction (TPR–H_2_). The TPR profile for the Ni/Al_2_O_3_ (Figure 2) catalyst showed two hydrogen consumption peaks. The first effect was observed at 320 °C and was related to the reduction of surface NiO species weakly bonded to the support. The second reduction peak, located in the temperature range of 380–810 °C, was due to the reduction of the Ni–O–Al species (as in the NiAl_2_O_4_ spinel) [22].

The TPR profile of Ni/CeO_2_-Al_2_O_3_ catalyst showed four distinctive peaks of reduction. The first and second reduction peaks were due to the reduction of NiO weakly interacting with the support and the reduction of the CeO_2_ surface to nonstoichiometric oxides CeO_2-x_ [23]. The third reduction step located on the TPR profile represents the reduction of NiO strongly interacting with the support. The last effect of hydrogen consumption is related to the reduction of the NiAl_2_O_4_ spinel [23]. The addition of CeO_2_ to the Al_2_O_3_ support shifted the reduction effects towards lower temperatures. The same results were also obtained by Wang et al. [24].

The reduction of 1%Ru-20%Ni/Al_2_O_3_ catalyst occurred similar to how it was observed for the monometallic Ni catalyst. The only difference was the appearance of the additional effect at temperatures below 200 °C [22,25]. This effect was explained by Li et al. as a reduction of the NiO-RuO_2_ alloy, which manifested as two overlapping effects on the TPR profile [26]. Thus, at a temperature of about 200 °C, RuO_2_ is reduced first, and then NiO. Comparing the TPR profile of the catalyst Ni/Al_2_O_3_ and Ru-Ni/Al_2_O_3_, there is a visible shift in NiO and NiAl_2_O_4_ reduction towards lower temperatures. It is well known that the promotion of the nickel catalyst by ruthenium facilitates its reduction, which confirms the shift in the reduction effect towards the lower temperature range. This is due to the spillover effect between ruthenium and nickel (II) oxide.

The TPR profile registered for 1%Ru-20%Ni/5%CeO_2_-95%Al_2_O_3_ showed four stages of reduction. The first stage with a maximum at 105 °C was related to the reduction of RuO_2_ [27]. The second effect observed at 280–380 °C was due to the reduction of free NiO. The third peak with a maximum at about 420 °C was related to the reduction of CeO_2_ present on the catalyst surface. The fourth step with a maximum at ~580 °C was due to the reduction of NiAl_2_O_4_, and the last effect, in the temperature range 700–800 °C was associated with the presence of CeO_2_ in mass.

### 3.4. TPR–CH_4_ Studies

**Figure 3:**
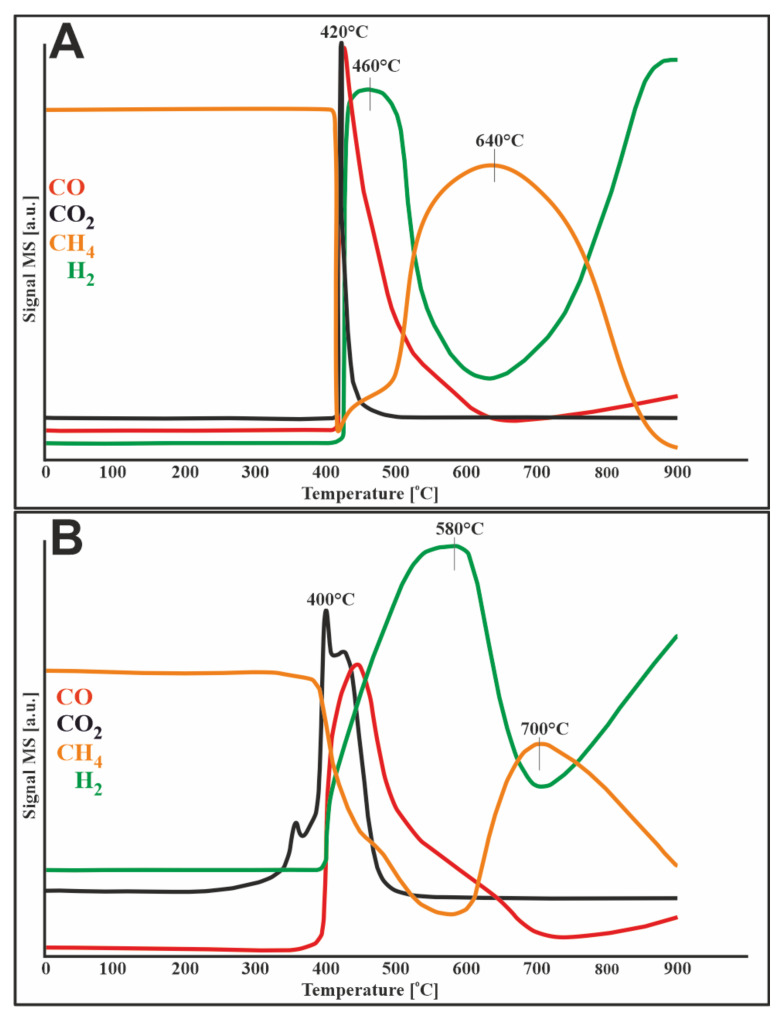
TPR-CH_4_ profiles of (**A**) 20%Ni/5%CeO_2_-95%Al_2_O_3_ and (**B**) 1%Ru-20%Ni/5%CeO_2_-95%Al_2_O_3_ catalysts.

Figure 3 shows the methane reduction profiles for two catalysts (20%Ni/5%CeO_2_-95%Al_2_O_3_, 1%Ru-20%Ni/5%CeO_2_-95%Al_2_O_3_). In both cases, the decomposition of methane started at a temperature of about 400 °C. The results of this process were the release of carbon oxides and hydrogen, which can be illustrated by the following reactions:

CH_4_ → C + 2H_2_ (11)

H_2_ + NiO → H_2_O + Ni and/or 2H_2_ + RuO_2_ => Ru + 2H_2_O (reduction of NiO and RuO_2_) (12)

H_2_O + CH_4_ → CO + 3H_2_ (methane steam reforming) (13)

CO + H_2_O ⇌ CO_2_ + H_2_ (water gas shift reaction) (14)

In the case of the bimetallic catalyst, CO_2_, which appeared before the methane decomposition process, originated from the oxidation of impurities on the catalytic surface.

In Figure 3A, in the temperature range of 400–900 °C, the evolution of methane is observed with the simultaneous loss of hydrogen and carbon oxides, which is attributed to the methanation process. The bimetallic catalyst (Figure 3B) behaves in the same way, except that the methanation starts later, at 600 °C.

### 3.5. Methane Decomposition and Carbon Monoxide Disproportionation

The thermal energy converts gaseous methane to hydrogen and solid carbon at high temperatures in the range from 1000 to 1200 °C. It is believed that methyl radicals formed during this process can polymerize and form cyclic and aromatic precursors to graphitic soot particles. The catalysts effectively shift the reaction towards much lower temperatures of ~400–500 °C by reducing the activation energy of the methane decomposition reaction. With a temperature increase of just above 800 °C, most active catalysts show a decrease in hydrogen production with a further increase in reactor temperature. Normally, such a decrease in activity is caused by catalyst deactivation.

**Figure 4:**
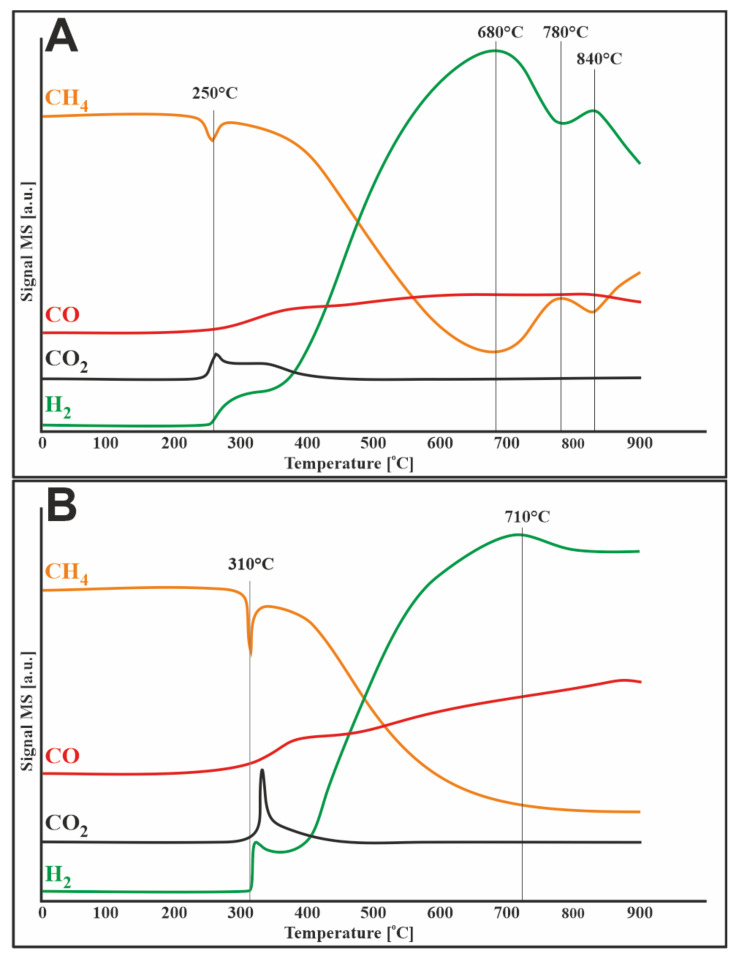
TPSR profile for methane decomposition over (**A**) 20%Ni/5%CeO_2_-95%Al_2_O_3_ and (**B**) 1%Ru-20%Ni/5%CeO_2_-95%Al_2_O_3_ catalysts.

To investigate the susceptibility of catalysts (20%Ni/5%CeO_2_-95%Al_2_O_3_, 1%Ru-20%Ni/5%CeO_2_-95%Al_2_O_3_) to carbon deposition TPSR measurements for methane decomposition and Boduard process were performed. The obtained results (Figure 4A) showed that methane started to decompose at about 250 °C over a monometallic catalyst. The addition of ruthenium to the catalytic system shifted the start of the process by 50 °C to higher temperatures. The observed uptake of methane was accompanied by the simultaneous release of carbon oxides. This can be due to several simultaneous processes, namely: (i) formation of carbon species on the surface of the catalyst due to the decomposition of methane and (ii) oxidation of these species to CO_2_, and then to CO by mobile oxygen in the lattice of CeO_2_.

Then, the reaction rate gradually increased along with the temperature increase to approx. 680 °C for the monometallic catalyst and approx. 710 °C for the bimetallic system. Then, a decrease in the reaction rate was observed for both catalysts. The 20%Ni/5%CeO_2_-95%Al_2_O_3_ system showed more rapid deactivation than the bimetallic catalyst, which was related to the difference in the rate of carbon deposition on the surface of catalysts.

Similar results were obtained by Jiang et al. [28], who observed that in the Ni/Al_2_O_3_ catalytic system without the addition of CeO_2_, the conversion of methane decreased at 850 °C. They attributed this to the aggregation of nickel particles into larger particles, which leads to a reduction in the active sites of methane decomposition. Decreasing the conversion of methane can also be explained by carbon deposition and hence catalyst deactivation. The addition of CeO_2,_ due to its oxygen storage and release (OSC) properties, oxidizes the formed carbon deposit, which results in the release of CO_2_ and CO shown in Figure 4. This theory is also confirmed by the TPR-H_2_ profiles (Figure 2), which show a reduction of CeO_2_, related to the release of oxygen, which oxidizes the carbon deposit formed during the decomposition of methane.

**Figure 5:**
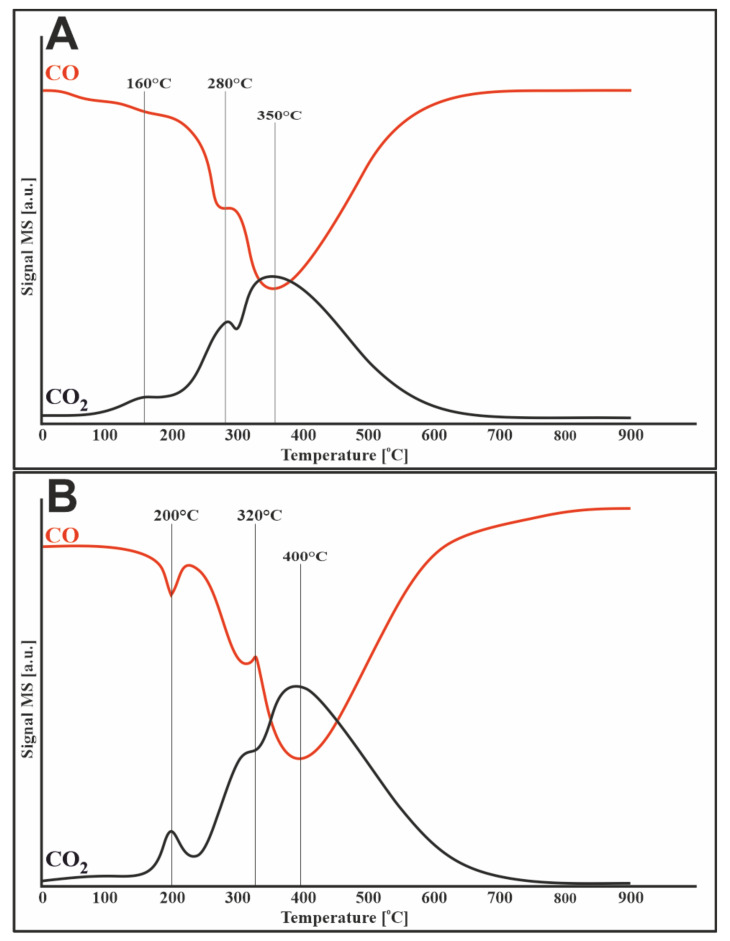
TPSR profile for CO disproportionation over (**A**) 20%Ni/5%CeO_2_-95%Al_2_O_3_ and (**B**) 1%Ru-20%Ni/5%CeO_2_-95%Al_2_O_3_ catalysts.

The second reaction that causes the catalyst to carbonize during the mixed methane reforming process is CO disproportionation (Figure 5). According to the TPSR results, the reaction takes place for both catalysts in a wide range from 50 to 700 °C. The maximum CO consumption rate was observed at 350 and 400 °C for the monometallic and bimetallic systems, respectively. At higher temperatures, the reaction did not proceed due to thermodynamic constraints. Therefore, CO disproportionation is the major pathway for carbon deposition below 500 °C. However, above this temperature, the decomposition of methane is solely responsible for the deposition of the carbon deposit.

### 3.6. Catalytic Activity Measurements/Total Organic Carbon (TOC) Analysis

Table 2 summarizes the conversions of carbon dioxide and methane, as well as the concentrations of carbon deposit on the surface of the catalyst after the reforming process and TPSRs with different gasification agents. The smallest amount of carbon deposits (1.5%) was formed on the bimetallic catalyst after reaction at 750 °C. On the other hand, the highest concentration of carbon deposit (4.6%) was observed on the surface of catalyst 20%Ni/CeO_2_-Al_2_O_3_ after the reaction at 700 °C. This can be explained by the lower catalytic activity and the high rate of the methane decomposition process (Figure 3A and Figure 5A).

Activity studies carried at various temperatures showed that the most active was the 1%Ru-20%Ni/CeO_2_-Al_2_O_3_ system, which showed a very high conversion of both CH_4_ and CO_2_ at 700 °C. Based on the obtained results, it can be seen that the addition of Ru to the active phase increased the conversion of the monometallic nickel catalyst. It is caused by the formation of Ru-Ni clusters, which leads to an increase in nickel dispersion on the surface and thus increases the number of active sites available for reaction gases [20,29]. Moreover, the Ru particles themselves may also serve as active centers in the mixed methane reforming process.

**Figure 6:**
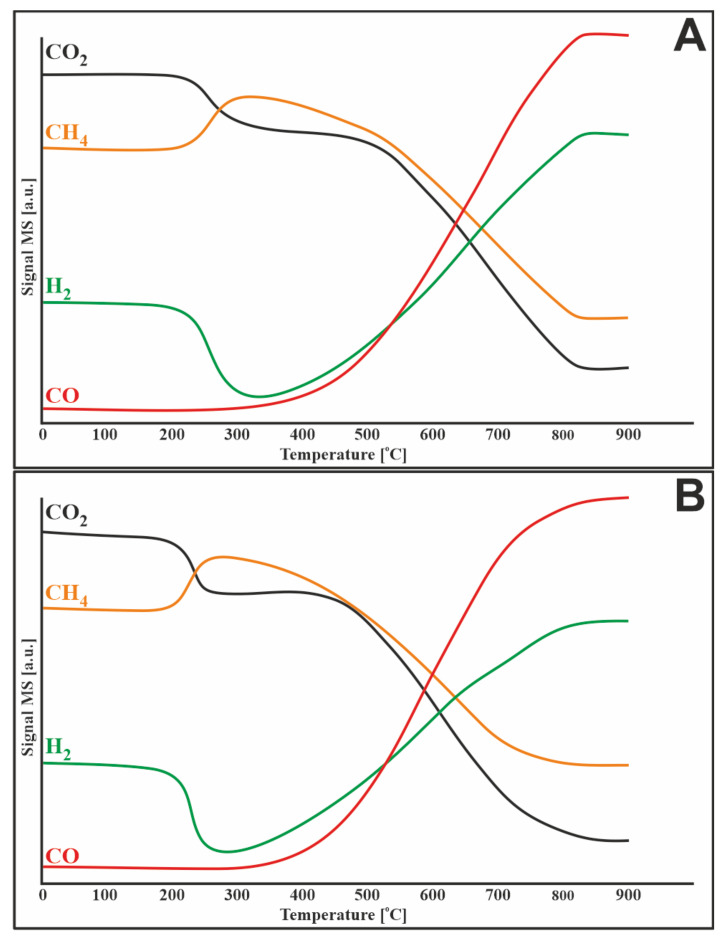
TPSR profile for mixed methane reforming over (**A**) 20%Ni/5%CeO_2_-95%Al_2_O_3_ and (**B**) 1%Ru-20%Ni/5%CeO_2_-95%Al_2_O_3_ catalysts.

The TPSR measurements for mixed reforming of methane process (Figure 6) showed that the methane reforming started at a temperature above 300 °C. Below this temperature, the concentration of carbon dioxide gradually decreased, followed by an increase in the concentration of carbon monoxide. This effect was accompanied by a significant decrease in hydrogen concentration and the release of methane in the same temperature range [30]. These effects are related to the initiated methanation process.

### 3.7. Reactivity of Carbon Deposit towards Different Gasifying Agents (O_2_, H_2_, CO_2_, and H_2_O)

The temperature-programmed surface reaction experiments were carried out to determine the forms and reactivity of the carbon deposit formed during the mixed methane reforming process. The following gasifying agents were used for the tests: hydrogen, water vapor, carbon dioxide, and oxygen, and the results of the tests performed are presented in the figures below.

#### 3.7.1. Oxygen

**Figure 7:**
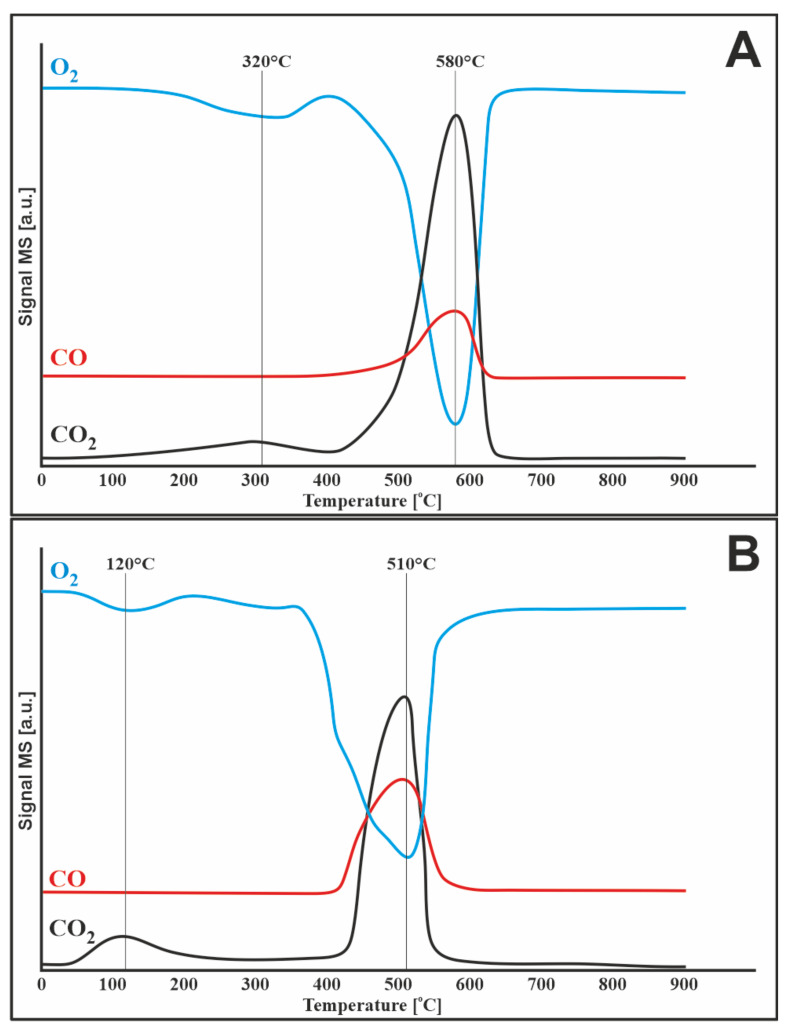
TPSR of oxygen (5%O_2_-95%Ar) stream with carbon deposited on the surface of the catalyst after mixed reforming of methane at 750 °C for (**A**) 20%Ni/5%CeO_2_-95%Al_2_O_3_ and (**B**) 1%Ru-20%Ni/5%CeO_2_-95%Al_2_O_3_.

The TPSR profile (Figure 7A) of the monometallic catalyst 20%Ni/CeO_2_-Al_2_O_3_ showed two effects of oxygen consumption, the first peak with a maximum at about 320 °C, and another significant peak with a maximum at about 580 °C. The latter effect was accompanied by a noticeable release of carbon oxides (greater effect on CO_2_ and less on CO), indicating oxidation of the inactive form of carbon deposit. On the other hand, for the first effect, there was no significant formation of carbon oxides. Therefore, the effect of oxygen consumption was likely related to the oxidation of negligible amounts of polymeric or encapsulated carbon followed by nickel particles. The addition of CeO_2_, due to its oxygen storage/transport (OSC) ability, led to easier oxidation of the carbon deposit than without cerium oxide (IV) [31].

The obtained results were confirmed by Wang in his work [24], in which he investigated the oxidation of carbon deposit on Ni/Al_2_O_3_ and Ni/CeO_2_-Al_2_O_3_ catalysts. It was found that the carbon removal in the systems containing cerium (IV) oxide began earlier than in the 20%Ni/Al_2_O_3_ systems. The profiles of the tested samples showed that the carbon deposit was more active for materials containing CeO_2_ than the Ni/Al_2_O_3_ catalyst.

#### 3.7.2. Hydrogen

The TPSR profile of a monometallic 20% Ni/CeO_2_-Al_2_O_3_ catalyst showed four distinctive curves related to carbon oxides, methane, and hydrogen. The green curve shows the two effects of hydrogen consumption. Both presented peaks are reflected in the other curves and are related to the evolution of mono- and carbon dioxide and methane, the same as in the case of the 20% Ni/Al_2_O_3_ system tested in the previous work [31]. The first effect, located at a temperature below 200 °C, is associated with the gasification of chemically adsorbed carbon, and the second, occurring at a temperature of about 550 °C, is attributed to the reduction of inactive carbon. In the same temperature ranges, the evolution of methane is visible, so these effects can be attributed to the methanation process. At lower temperatures, methane is likely formed by the reduction of carbon dioxide previously adsorbed during the reaction and/or formed by the oxidation of active carbon species by adsorbed water. It can be concluded that the catalyst with the addition of cerium (IV) oxide showed a shift in the carbon reduction effects towards lower temperatures compared to the 20% Ni/Al_2_O_3_ catalyst discussed in the previous work [31], which proves easier removal of the carbon deposit from the catalyst surface.

**Figure 8:**
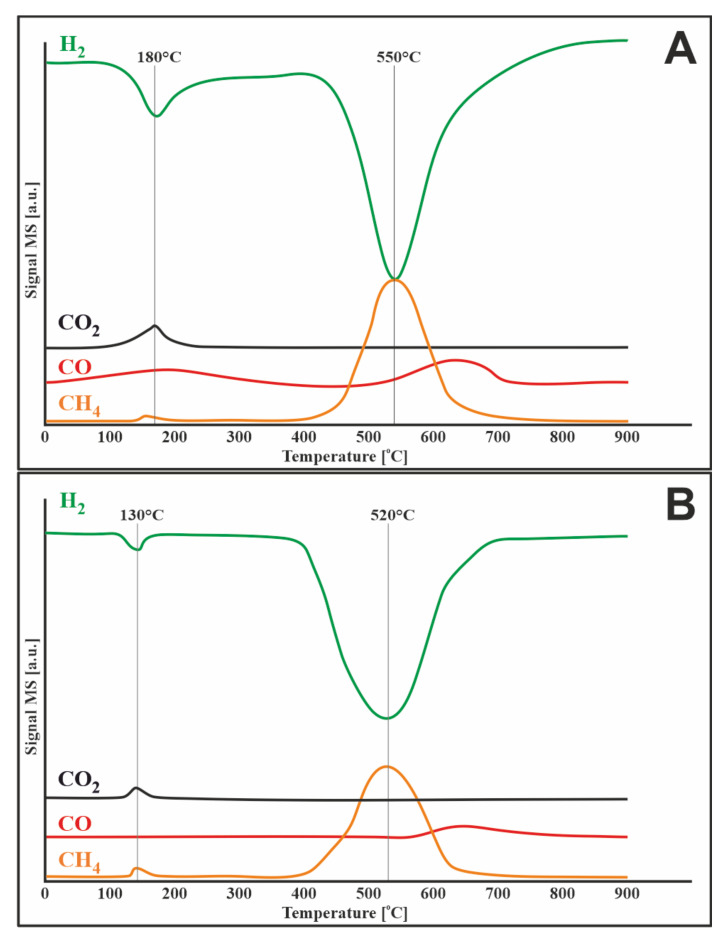
TPSR of pure hydrogen with carbon deposited on the surface of the catalyst after mixed reforming of methane at 750 °C for (**A**) 20%Ni/5%CeO_2_-95%Al_2_O_3_ and (**B**) 1%Ru-20%Ni/5%CeO_2_-95%Al_2_O_3_.

Figure 8B shows the TPSR profile for the 1%Ru-20% Ni/CeO_2_-Al_2_O_3_ catalyst. The bimetallic system behaves very similarly to the monometallic catalyst described above. The only noticeable difference is the shift in the effects on all curves towards lower temperatures, which is related to the addition of Ru to the active phase of the catalyst.

#### 3.7.3. Carbon Dioxide

**Figure 9:**
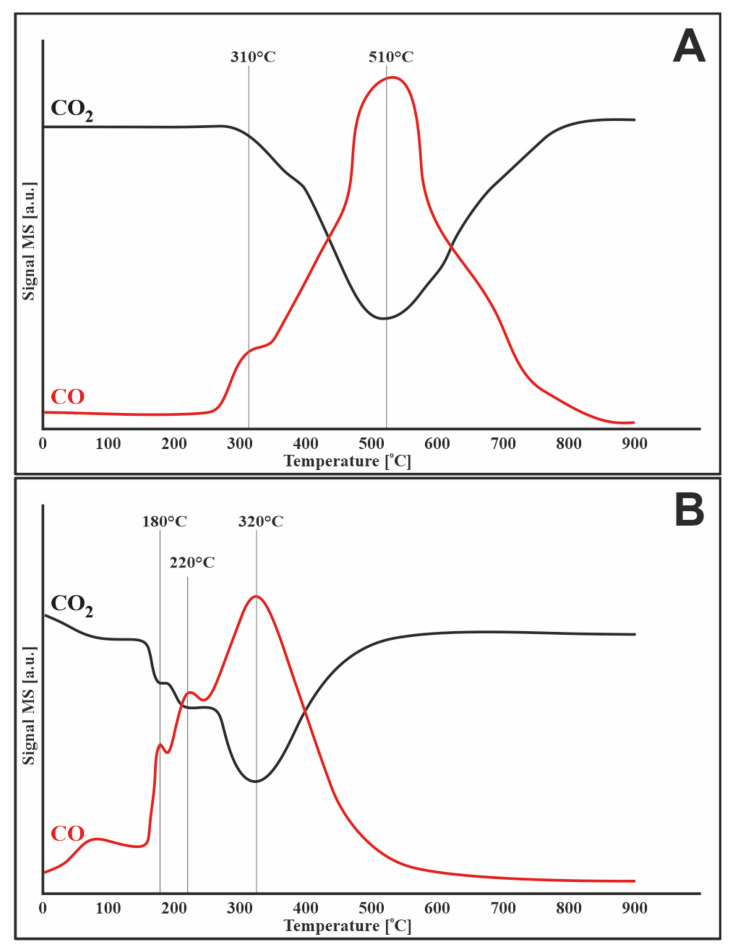
TPSR of carbon dioxide (5%CO_2_-95%Ar) stream with carbon deposited on the surface of the catalyst after mixed reforming of methane at 750 °C for (**A**) 20%Ni/5%CeO_2_-95%Al_2_O_3_ and (**B**) 1%Ru-20%Ni/5%CeO_2_-95%Al_2_O_3_.

Figure 9 shows the process of oxidation of the carbon deposit with carbon dioxide. The black curve shows the consumption of CO_2_, while the red curve is related to the evolution of CO. Figure 9A presents the results obtained for a monometallic sample of a catalytic material with support promoted with cerium (IV) oxide. Compared to 20%Ni/Al_2_O_3_ from previous work [31], there is a clear shift in the oxidation process of the carbon deposit towards lower temperatures up to 510 °C. On the other hand, the TPSR profile for the bimetallic catalyst, which is shown in Figure 9B, showed an even lower temperature at which the carbon removal process with a maximum at 320 °C. As can be seen, the addition of CeO_2_ and/or ruthenium resulted in significant changes in the TPSR profiles.

Additionally, the presented TPSR profiles (Figure 9) are characterized by a two- (Figure 8A) and three-stage (Figure 9B) process of oxidation of the carbon deposit. This may be due to the presence of oxygen vacancies in CeO_2_. The released electrons from cerium are transferred to the center of the Ni atom, increasing the density of the Ni electron cloud, allowing the catalyst to absorb more amount of CO_2_ and promote the formation of CO_2_, which can oxidize the carbon deposit [32].

#### 3.7.4. Water Vapor

**Figure 10:**
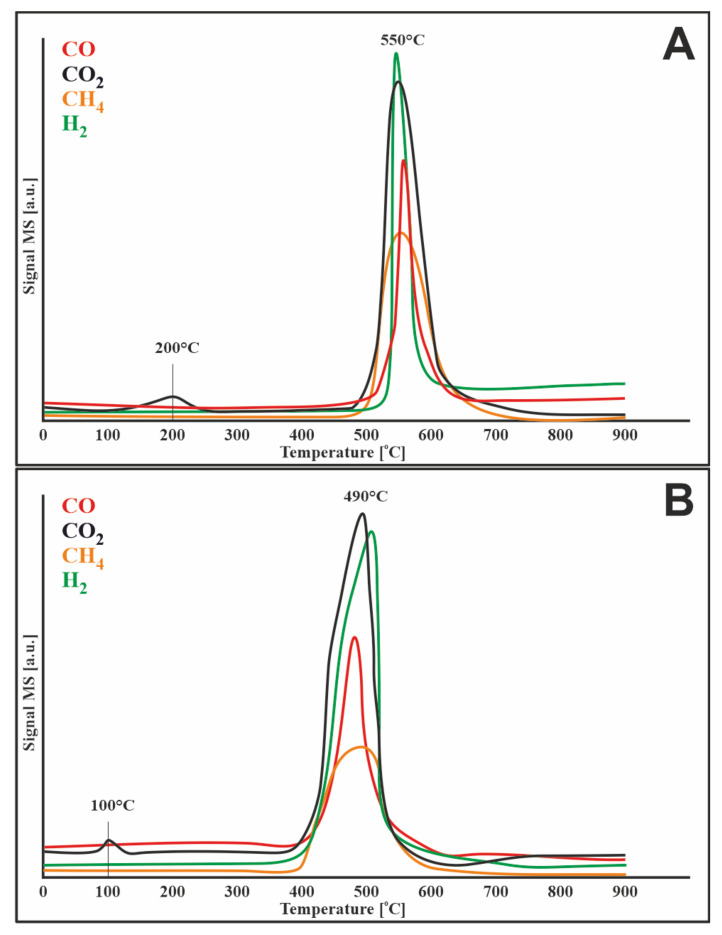
TPSR of water vapor (5%H_2_O-95%Ar) steam with carbon deposited on the surface of the catalyst after mixed reforming of methane at 750 °C for (**A**) 20%Ni/5%CeO_2_-95%Al_2_O_3_ and (**B**) 1%Ru-20%Ni/5%CeO_2_-95%Al_2_O_3_.

Figure 10 shows the TPSR profiles for the oxidation of the carbon deposit by the water vapor. Results obtained for the monometallic catalyst 20%Ni/5%CeO_2_-95%Al_2_O_3_ are exhibited in Figure 10A. The profile shows four curves related to the evolution of hydrogen, methane, and carbon oxides in the temperature range 490–700 °C. Carbon monoxide, carbon dioxide, and hydrogen were generated by gasification of the carbon deposit with steam according to Reactions (4) and (5). The formed H_2_ and CO_2_ also oxidize the carbon on the catalyst surface (Equations (3) and (6), which results in the formation of methane in the case of gasification of the carbon deposit with hydrogen and carbon monoxide if it was CO_2_.

Compared to the nickel catalyst tested in the previous work [31], the catalyst with the addition of cerium (IV) oxide showed a narrower temperature range for the oxidation of the carbon deposit. There is also a visible shift in the starting temperature of the process from 430 to 490 °C by modification with cerium (IV) oxide [31]. On the other hand, the 1%Ru-20%Ni/CeO_2_-Al_2_O_3_ catalyst showed lower temperatures for removing the carbon deposit from the catalytic surface (Figure 10B). This phenomenon was caused by the addition of ruthenium to the active phase of the catalyst.

Both catalyst systems were tested after the TPSR processes using the TOC technique, and the results are presented in Table 2. As can be seen, the water vapor completely removed the carbon deposit from both carbonized catalytic samples. The catalyst 1%Ru-20%Ni/5%CeO_2_-95%Al_2_O_3_ was regenerated the fastest. In this case, cerium (IV) oxide, along with ruthenium showed a promotional effect on the speed and efficiency of carbon deposit removal.

Walker reported that the rate of carbon deposit removal by gasifying agents is as follows: O_2_ (10^5^) > H_2_O (3) > CO_2_ (1) > H_2_ (3 × 10^−3^) [33]. The relative rates of uncatalyzed gasification are shown in brackets. In this case, oxygen is the most effective agent. However, its use causes the reaction to be highly exothermic, which can lead to rapid deactivation of the catalyst by sintering. Since the feed gas does not contain oxygen in mixed methane reforming, steam is the most effective agent for removing carbon deposits.

## 4. Influence of Ruthenium on the MRM Process

The results obtained from the above research techniques and from the study of catalytic activity, as well as from the review of the scientific literature, lead to the following assumptions of the mechanism of the mixed methane reforming process [34]:

1. The addition of ruthenium causes the formation of Ru-CH_x_ sites on support. (Equation (15)) [34].
2. Oxidation of Ru with activated CO_2_ results in the formation of Ru-O and Ru-CO species (Equations (16) and (17)). Ru-O can also originate from the dissociation of water vapor (with the formation of hydrogen, Equation (18)). This can take place directly through dissociation on the metal particles (Equation (18)) or indirectly through the support (Equations (19)–(22)) [34].
3. Transfer of oxygen from Ru-O to Ru-CH_x_ with the formation of further CO, CO_2_, and H_2_ (Equations (23)–(25)). As a consequence, Ru is regenerated (Equations (24) and (25)) [34].
4. The adsorbed hydrogen (Ru-H) can also combine with the adsorbed oxygen (Ru-O) to form a hydroxyl group and then combine with the hydrogen to form water vapor (H_2_O(g)) which then desorbs from the ruthenium surface onto effluent gas mixture (Equations (26) and (27)) [34].

2Ru + CH_4_ → Ru-CH_x_ + Ru-H (15)

Ru + CO_2_ → Ru-CO_2_ (16)

Ru + Ru-CO_2_ → Ru-CO + Ru-O (17)

Ru + H_2_O → Ru-O + H_2_(g) (18)

H_2_O + S → S-H_2_O S—symbolizes an empty site on the support (19)

S-H_2_O + 2Ru → Ru-H + Ru-OH (20)

Ru-OH + Ru-OH → Ru-O + H_2_O(g) + Ru (21)

H_2_ + Ru-O → H_2_O(g) +Ru (22)

Ru-CH_x_ + Ru-O → Ru-CO/Ru-CO_2_ or Ru-CH_2_O/Ru-CHO + Ru-H (23)

Ru-CO → CO(g) + Ru(24)

2Ru-H → H_2_(g) + 2Ru (25)

Ru-H + Ru-O → Ru-OH + Ru(26)

Ru-H +Ru-OH → H_2_O(g) + 2Ru (27)

## 5. Role of Cerium Oxide the MRM Process

**Figure 11:**
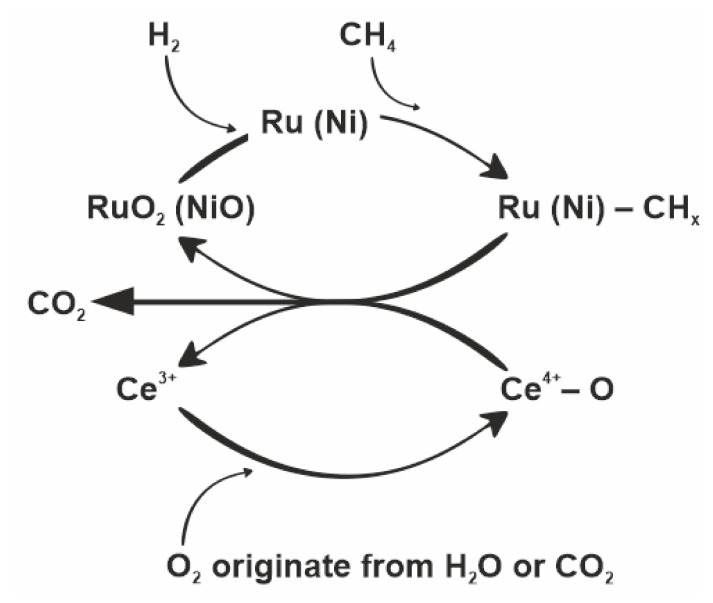
Diagram of oxygen transport during mixed methane reforming on the catalyst (Ru)-Ni/Al_2_O_3_-CeO_2_.

Kurungot and Yamaguchi investigated in their work the active role of the support in methane reforming [35] for the CeO_2_-doped Rh/γ-Al_2_O_3_ catalyst. They suggested that better catalyst performance could be obtained due to the kinetic and oxidative stabilization of the catalyst matrix with CeO_2_. The Mars–van Krevelen redox cycle (similar to Figure 11) can be proposed as a possible mechanism.

The adsorbed methane reduces the metal oxide, which is reoxidized by the oxygen from the feed. The rate of methane adsorption can be significantly reduced by the loss of oxygen from the system. This can be prevented by replenishing oxygen as a result of the addition of cerium (IV) oxide, which can accelerate the transport of oxygen in the system due to its ability to store and release oxygen. The described property of cerium is possible due to the stability of the forms: Ce^3+^ and Ce^4+^, enabling the oxygen transfer between CeO_2_ and nonstoichiometric oxides such as CeO_2−x_. The lattice oxygen released during cerium reduction can react with CH_4_ and CO.

A similar mechanism for Ni/Ce_x_Zr_1−x_O_2_ was described by Dong et al. [36]. They showed that CeO_x_ improves the dissociation of H_2_O and accelerates the reaction of water vapor with active sites on the nickel surface near the interface between the metal and the support, thus reducing carbon deposit deposition and promoting catalyst stability during the methane reforming process. The mechanism proposed by Dong describes the adsorption of individual components of the feed gas on the surface of the catalytic system. While methane dissociates on the nickel surface, water and oxygen competitively adsorb on the nickel and the Ce_x_Zr_1−x_O_2_ support. It is assumed that the catalyst surface is mostly occupied by C, CH_x_, O, and OH. The authors conclude that the high activity and stability of 15% by weight of Ni on CexZr_1−x_O_2_ is mainly due to a good balance between active centers (some are related to the activation of methane, and others are used to activate water vapor or oxygen).

The mechanism based on cerium (IV) oxide, which can be proposed in the mixed methane reforming process, was shown in Figure 11. This is also the Mars–van Krevelen redox cycle similar to that proposed by Kurungot, but with a few differences. The first noticeable difference is the metal oxide reducing agent, which in the mixed methane reforming process was hydrogen from the feed gas. On the other hand, in the work of Kurungot and Yamaguchi, the hydrogen that reduced the metal oxide originated from the decomposition of methane and appeared at higher temperatures. In connection with TPR-CH_4_ studies, it can be seen that methane began to decompose at 400 °C (Figure 3). Comparing TPR-CH_4_ results with the data obtained during TPSR mixed methane reforming (Figure 6), it can be seen that the decomposition of methane (in the MRM process) started faster, at about 300 °C, since metal oxides were already reduced by hydrogen from the feed gas, and methane may have been decomposed through dissociative adsorption on nickel particles by a series of dehydrogenation steps leading to elemental carbon and another hydrogen. The second difference between the mechanism described by Kurungot and the one proposed in this work is the source of oxygen causing oxidation of the reduced CeO_2_. The feed gas of the MRM process contains water vapor and carbon dioxide, which were the source of atomic oxygen formed as a result of H_2_O and CO_2_ dissociative decomposition. The oxygen obtained in this way can be used to oxidize Ce^3+^ to Ce^4+^.

## 6. Summary

Mixed reforming of methane was studied using (Ru)-Ni catalysts supported on CeO_2_-Al_2_O_3_. The implementation of Ru and CeO_2_ in the catalyst played a significant role in reactivity toward deposited carbon. Among the all catalysts tested, the Ru-Ni/CeO_2_-Al_2_O_3_ catalyst exhibited better catalytic activity in the mixed methane reforming process. In addition, the strong oxygen storage and release properties of CeO_2_ led to less carbon deposition and thereby higher stability for nickel-based catalysts. Regardless of the gasifying agent used, the carbon deposit was removed from the surface of the catalytic system. Water vapor was found to be the most efficient in removing the carbon deposit.

## Figures and Tables

**Table 1:** Catalysts and support properties.

Sample	Metal Content in the Catalyst	Average Size of Ni Crystallites (nm)	Specific Surface Area (m^2^/g)	Pore Size (nm)
5%CeO_2_-95%Al_2_O_3_	-	-	170	1.9–2.3
20%Ni/5%CeO_2_-95%Al_2_O_3_	18.2% Ni	45	147	2.6–3.6
1%Ru-20%Ni/5%CeO_2_-95%Al_2_O_3_	18.8% Ni	16	149	2.5–3.6

**Table 2:** CH_4_ and CO_2_ conversions for mono- and bimetallic catalyst systems with the amount of carbon deposit on the surface of catalysts after mixed methane reforming and after gasification during TPSR.

Catalytic System/Sample Carbonization Temperature (°C)	Conversion after 3 h Reaction		Carbon Content (%) after:				
	CH_4_ (%)	CO_2_ (%)	MRM Process	TPSR (5%H_2_-95%Ar)	TPSR (99.99%H_2_)	TPSR (5%CO_2_-95%Ar)	TPSR (5%H_2_O-95%Ar)
20%Ni/CeO_2_-Al_2_O_3_ [650]	44	44	2.3	0	0	0	0
20%Ni/CeO_2_-Al_2_O_3_ [700]	68	62	4.6	0	0	0	0
20%Ni/CeO_2_-Al_2_O_3_ [750]	78	71	3.5	0	0	0	0
1%Ru-20%Ni/CeO_2_-Al_2_O_3_ [650]	57	54	1.7	0	0	0	0
1%Ru-20%Ni/CeO_2_-Al_2_O_3_ [700]	88	84	3.5	0	0	0	0
1%Ru-20%Ni/CeO_2_-Al_2_O_3_ [750]	93	86	1.5	0	0	0	0

## Data Availability

The data presented in this study are available on request from the corresponding author.
